# Complete mitochondrial DNA genome of grey pomfret *Pampus cinereus* (Bloch, 1795) (Perciformes: Stromateidae)

**DOI:** 10.1080/23802359.2018.1481788

**Published:** 2018-06-12

**Authors:** Shouqiang Wang, Linlin Zhao, Yuan Li, Zhaohui Zhang, Zongling Wang, Tianxiang Gao

**Affiliations:** a College of Environmental Scinence and Engineering, Ocean University of China, Qingdao, P.R. China;; b The First Institute of Oceanography, SOA, Qingdao, P.R. China;; c Third Institute of Oceanography, SOA, Xiamen, P.R. China;; d Fishery College, Zhejiang Ocean University, Zhoushan, P.R. China

**Keywords:** Stromateidae, mitochondrial genome, *Pampus cinereus*

## Abstract

In this study, we sequenced the complete mitochondrial genome of *Pampus cinereus* (Bloch, 1795). This mitochondrial genome, consisting of 16,540 base pairs (bp), contains 13 protein-coding genes, two ribosomal RNAs, 22 transfer RNAs, and two mainly noncoding regions (control region and origin of light-strand replication) as those found in other vertebrates. Control region with 846 bp in length, is located between tRNA^Pro^ and tRNA^Phe^. The overall base composition of the heavy strand shows 27.4% of T, 27.5% of C, 30.0% of A, and 15.2% of G, with a slight A + T rich bias (57.4%). The complete mitochondrial genome data will provide useful genetic markers for the studies on the molecular identification, population genetics, phylogenetic analysis and conservation genetics.

The grey pomfret, *Pampus cinereus* (Bloch, 1795) is a common species distributed mainly in the Indo-Western Pacific. This species is characterized by a greatly extended anal fin and notably long pectoral fins. However, some previous reports considered *P*. *cinereus* as a synonym of *P*. *argenteus* (Haedrich 1967; Parin and Piotrovsky 2004; Cui et al. 2011). To clarify the misidentification of this species, sufficient genetic information is needed and helpful for rational utilization of *P. cinereus* resources. In this study, we present the complete mitochondrial genome of *P. cinereus* and reconstruct phylogenetic topology of *Pampus* species to demonstrate the taxonomic status of *P. cinereus*.

The sample of *P. cinereus* was collected from the coastal waters of Xiamen (24.40°N, 118.16°E) during May 2012 and deposited in Fishery Ecology Laboratory of Ocean University of China (OUC). The complete *P. cinereus* mitogenome was amplified using a long-PCR technique, and then subsequent sequencing was accomplished by primer walking method (Miya and Nishida 1999). The mitogenome sequence has been deposited in GenBank with accession number MH037008.

The complete mitochondrial genome of *P. cinereus* (16,540 bp in length) consists of 13 protein-coding genes, 22 tRNA genes, two rRNA genes, and two mainly non-coding control region (control region and origin of light-strand replication). Arrangement of all genes is identical to that of most vertebrates (Wang et al. 2008; Chen 2013; Chiang et al. 2013). Most of the genes are encoded on the heavy strand (H-strand), except for the eight tRNA genes (-Gln, -Ala, -Asn, -Cys,-Tyr, -Ser, -Glu, and -Pro), one non-coding region (origin of light-strand replication, O_L_) and one protein-coding gene (ND6). The overall base composition is 27.4% for T, 27.5% for C, 30.0% for A, and 15.2% for G, with a slight A + T-rich feature (57.4%) just as other marine fishes (Cui et al. 2009; Cheng et al. 2012). Except for COI, ND4, and ND6 starting with GTG, the remaining 10 protein-coding genes start with ATG. Five protein-coding genes are inferred to terminate with incomplete stop codons (ND2, COII, COIII, ND3, ND4), with five (ATPase8, ATPase6, ND4L, ND5, and Cyt *b*) sharing TAA and three (ND1, COI, ND6) using TAG as stop codons. These features are common among vertebrate mitochondrial genome, and TAA is supposed to be appeared via posttranscriptional polyadenylation (Anderson et al. 1981).

Phylogenetic relationship was constructed using NJ algorithm among six *Pampus* species based on 12 H-strand mitochondrial protein-coding genes, 22 tRNA and two rRNA genes (Figure 1). These sequences were aligned using the DNASTAR software. MEGA 5.0 (Tamura et al. 2011) was then used to construct neighbour-joining (NJ) tree based on Kimura 2-parameter (K2P) model. *Peprilus triacanthus* (Perciformes: Stromateidae) was used as outgroup. Numbers above branches indicate neighbour-joining bootstrap percentages. Only bootstrap values of >50% are shown in the above NJ tree. This phylogenetic tree shows that these six *Pampus* species were obviously different from each other and *P. cinereus* is more closely related to *P. argenteus* than other species of *Pampus* species.

**Figure 1.**
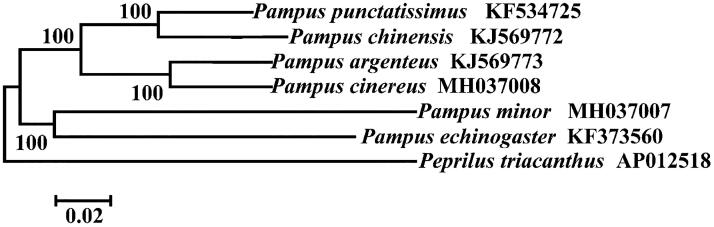
Phylogenetic relationship using NJ algorithm among *Pampus* species based on 12 H-strand mitochondrial protein-coding genes, 22 tRNA, and two rRNA genes. *Peprilus triacanthus* (Perciformes: Stromateidae) was used as outgroup.
